# Fentanyl and its derivatives: Pain-killers or man-killers?

**DOI:** 10.1016/j.heliyon.2024.e28795

**Published:** 2024-03-28

**Authors:** Jiri Patocka, Wenda Wu, Patrik Oleksak, Romana Jelinkova, Eugenie Nepovimova, Lenka Spicanova, Pavlina Springerova, Suliman Alomar, Miao Long, Kamil Kuca

**Affiliations:** a Department of Chemistry, Faculty of Science, University of Hradec Kralove, 50003 Hradec Kralove, Czech Republic; b School of Food and Biological Engineering, Hefei University of Technology, Hefei 230009, China; c NBC Defence Institute, University of Defence, 68201 Vyskov, Czech Republic; d Philosophical Faculty, University of Hradec Kralove, 50003 Hradec Kralove, Czech Republic; e Doping Research Chair, Zoology Department, College of Science, King Saud University, Riyadh-11451, Kingdom of Saudi Arabia; f Key Laboratory of Zoonosis of Liaoning Province, College of Animal Science & Veterinary Medicine, Shenyang Agricultural University, Shenyang 110866, China; g Biomedical Research Centre, University Hospital in Hradec Kralove, Sokolska 581, 50005 Hradec Kralove, Czech Republic

**Keywords:** Fentanyl, Fentanyl derivatives, Drug overdose, Pharmacology, Toxicology, Opioid epidemic

## Abstract

Fentanyl is a synthetic μ-opioid receptor agonist approved to treat severe to moderate pain with faster onset of action and about 100 times more potent than morphine. Over last two decades, abuse of fentanyl and its derivatives has an increased trend, globally. Currently, the United States (US) faces the most serious situation related to fentanyl overdose, commonly referred to as the opioid epidemic. Nowadays, fentanyl is considered as the number one cause of death for adults aged 18–45 in the US. Synthesis and derivatization of fentanyl is inexpensive to manufacture and easily achievable. Indeed, more than 1400 fentanyl derivatives have been described in the scientific literature and patents. In addition, accessibility and efficacy of fentanyl and its derivatives can play a potential role in misuse of these compounds as a chemical weapon. In this review, the properties, general pharmacology, and overdose death cases associated with fentanyl and selected derivatives are presented. Moreover, current opioid epidemic in the US, Moscow theatre hostage crisis, and potential misuse of fentanyl and its derivatives as a chemical weapon are disclosed.

## Highlights

• The review summarizes current information about fentanyl and its notable known derivatives.
• Abuses and accidents associated with fentanyl and derivatives are disclosed in the review involving current opioid epidemic and possible future misuse of the compounds.
• Data regarding overdose deaths in the US and Europe caused by highlighted fentanyls are consistently described in the review.
• Overall drug overdose deaths in the US between 1999 and 2021 are compared with overall overdose deaths caused by fentanyl and derivatives.

## 1 Introduction

Fentanyl is a synthetic opioid analgesic, utilized as both painkiller and anesthetic [1]. However, fentanyl and its derivatives are used illegally in many countries and due to their high efficacy and toxicity cases of intoxication and fatal poisoning increasing globally [2]. In 2021, 71 074 deaths of overall 107 521 drug overdose death cases were associated with synthetic opioids, in the US [3]. Fentanyl and fentanyls, its synthetic derivatives, are illicitly marketed also in many European countries. The drugs are often sold as a heroin replacement. Adverse effects of fentanyls are similar to those caused by heroin and other opioids, including drowsiness, addiction, bradycardia, respiratory depression, unconsciousness and others. Serious cases of overdose can result in coma and/or death [4]. Abuse of fentanyl/s is not restricted only to the US and Europe, but becomes a worldwide problem [[5], [6], [7]]. Illicitly synthesized or “designer fentanyls”, occasionally also referred as non-pharmaceutical fentanyls have recently appeared in the illegal market. Designer fentanyls are usually misused as less expensive replacements of heroin, due to less expensive starting materials and equipment required for their preparation [4]. As the result of easily achievable production and modification, different fentanyls with variable biological effects can be prepared. Therefore, there are dozens of extremely toxic fentanyls with unknown properties on the illegal drug market.

## 2 Fentanyl

### 2.1 Physico-chemical properties of fentanyl

Fentanyl (CAS Registry Number: 437-38-7), according to the International Union of Pure and Applied Chemistry (IUPAC) nomenclature also known as *N*-phenyl-*N*-[1-(2-phenylethyl)piperidin-4-yl]propenamide, with chemical formula C_22_H_28_N_2_O has molecular mass of 336.479 g/mol. It is a white crystalline powder with melting point of 87.5 °C. Solubility of the compound in water is limited (0.2 mg/mL at 25 °C) [8,9]. Fentanyl is a lipophilic compound (log*P* 2.3 octanol/buffer pH 7.4) [10] which can easily enter the central nervous system (CNS) [11]. The pKa of fentanyl is 8.44 at 25 °C and it is estimated that under normal homeostatic conditions, pH 7.4 and temperature of 37 °C, about 16% of fentanyl in blood is unionized [12] thus it can bound to erythrocytes, albumins, and other endogenous compounds [11]. Fentanyl is commercially available as a citrate salt that requires no preservatives [13].

### 2.2 Synthesis of fentanyl

**Fig. 1:**
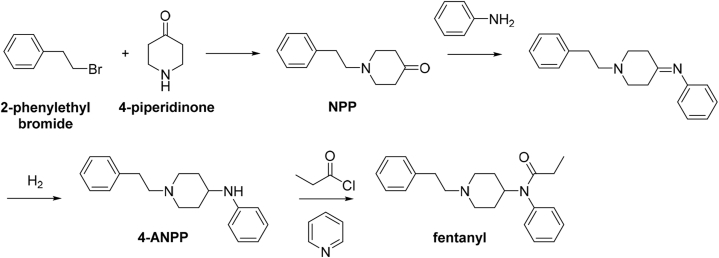
**Synthesis of fentanyl.** Simplified reaction scheme as reported by Ref. [15].

Fentanyl was for the first time synthesized in 1959 by Janssen Pharmaceuticals, a pharmaceutical company founded by Paul Janssen in Belgium. The drug has been developed in order to find rapid and effective analgesic. At that time, fentanyl was the most lipophilic and effective opioid drug, which was about 100 times stronger than morphine with faster onset of action [14]. However, Janssen method requires experienced laboratory worker and an expensive palladium carbon reagent. In 1980s, an entirely novel synthetic route has been developed based on the reaction of 4-piperidinone with 2-phenylethyl bromide providing *N*-phenethyl-4-piperidinone (NPP). In the second reaction step, NPP reacts with aniline to give *N*-phenyl-1-(2-phenylethyl)-4-piperidinimine, which upon reduction of imine group provides 4-anilino-*N*-phenethyl-piperidine (4-ANPP). As the final step of this reaction sequence, a secondary amine of 4-ANPP is acylated under conditions with propionyl chloride to give desired fentanyl (Fig. 1) [15]. This reaction sequence represents an effective preparation method without requirements of professional synthetic experiences or laboratory equipment. All materials and equipment can be easily purchased from the internet. These facts facilitate illegal production of fentanyl and allow its further derivatization.

### 2.3 Pharmacology of fentanyl

#### 2.3.1 Structure and types of opioid receptors

Opioid receptors are G protein-coupled receptors (GPCRs) that act as mediators of various stimuli from different hormones, neurotransmitters or drugs. All GPCRs consist of seven membrane-spanning α-helical segments with intracellular and extracellular loop regions. The receptors involve intracellular C-terminus and extracellular N-terminus. Individual GPCRs have unique combination of activities for signal transduction involving multiple G protein subtypes, G protein-independent signaling pathways and complex regulatory processes [16]. They are distributed widely in the nervous system, but also in peripheral organs such as heart, lungs, liver, as well as in gastrointestinal and reproductive tracts. Their distribution significantly varies among organs and among different animal species. Opioid receptors are essential to provide various physiological functions such as pain modulation, mood, locomotion, thermoregulation, diuresis, and stress. The GPCRs play a significant role in regulation of respiratory, gastrointestinal and cardiovascular systems. However, abuse of opioid compounds causes an addiction which affects nervous system and homeostasis of the body [17]. In general, opioids are divided into two categories including endogenous opioids (endorphins, enkephalins, endomorphins, dynorphins, and nociceptin/orphanin) and exogenous opioids (morphine, heroin, fentanyl and others). Representants of both categories bind to the same receptors [18]. To date, four types of opioid receptors are known. Three classical opioid receptors; represented by μ-opioid peptide (MOP) receptors, δ-opioid peptide (DOP) receptors, and κ-opioid peptide (KOP) receptors; are complemented by non-classical nociception opioid peptide (NOP) receptor. Above-mentioned abbreviations of receptors are proposed by Nomenclature Committee of the International Union of Basic and Clinical Pharmacology (NC-IUPHAR) [19]. Within four main types of opioid peptide receptors several receptor subtypes exist: MOP receptors include μ1, μ2, and μ3 receptors; DOP receptors include δ1, and δ2 receptors; KOP receptors include κ1, κ2, and κ3 receptors. Various endogenous opioids bind to different types and subtypes of opioid receptors resulting in distinct physiological functions. The μ1 receptor is responsible for analgesia and dependence; the μ2 receptor plays a role in euphoria, respiratory depression, miosis, decreased motility of gastrointestinal tract, and dependence; the μ3 receptor causes vasodilation. DOP receptors are associated with analgesia and reduced gastric motility; KOP receptors play a role in analgesia, diuresis, and dysphoria; NOP receptor associates with analgesia and hyperalgesia [18]. Fentanyl is a potent MOP receptor agonist, however it also exhibits lowered affinity to KOP and DOP receptors [20]. Inhibitory constant (Ki) of fentanyl for MOP receptor is 0.135 nM, for KOP receptor is 174 nM, and for DOP receptor is 220 nM [21].

#### 2.3.2 Signaling pathways of MOP receptors

Opioids that are commonly used for pain modulation act on MOP receptors. These opioids generally belong to effective analgesics; however, they are also efficient mood enhancers. They cause activation of central dopamine reward pathways that modulate euphoria [22]. Activation of MOP receptors by ligands provides different signaling such as G protein signaling pathway and β-arrestin signaling pathway [23]. The G protein signaling pathway is mediated via G proteins involving G_αi/o_ and G_αz_ classes that are coupled with G_β_, and G_γ_ subunits. Before ligand binding to MOP receptor, guanosine diphosphate (GDP) is bound to G_α_ subunit of heterotrimer G_αβγ_. However, binding of ligand to the receptor provides a conformational change, an activation of the receptor, which results in exchange of GDP, bound to G_α_ subunit, to guanosine triphosphate (GTP). Subsequently G_αβγ_ heterotrimer fractionates to G_α_ subunit bearing GTP and heterodimer G_βγ_. Subunit G_α_ inhibits adenylyl cyclase which causes reduced production of cyclic adenosine monophosphate (cAMP) which in turn affects membrane repolarization [[24], [25], [26]]. Heterodimer G_βγ_ inhibits voltage-gated calcium channels (VGCCs) which results in reduced transmitter release in neurons and activates G protein coupled inwardly-rectifying potassium channels (GIRKs) causing hyperpolarization in neurons [26,27]. Thus, G proteins are incorporated in the analgesic effect of MOP receptors [24]. On the other hand, agonist of MOP receptor can also promote β-arrestin signaling pathway. This activation results in G protein-coupled receptor kinases (GRKs)-mediated phosphorylation of serine and threonine residues located on the C-terminus or intracellular loops of the receptor. However, the phosphorylation depends on different factors such as single point mutations in MOP receptor or type of opioid agonist. Therefore, various opioid agonists reveal different profile of phosphorylation. The phosphorylation results in recruitment of β-arrestins that provide subsequent responses [[28], [29], [30]]. This signaling pathway promotes receptor internalization and desensitization that may lead to adverse effects such as respiratory depression and receptor desensitization [31]. Experiments in β-arrestin 2 knockout mice revealed preserved and potentiated analgesic effect of morphine [32]. Another study revealed that β-arrestin 2 knockout mice expressed higher protection against morphine-induced constipation and respiratory depression [33]. These studies supported hypothesis that β-arrestin signaling pathway, in comparison to G protein signaling pathway, contributes more to adverse opioid effects [27].

**Fig. 2:**
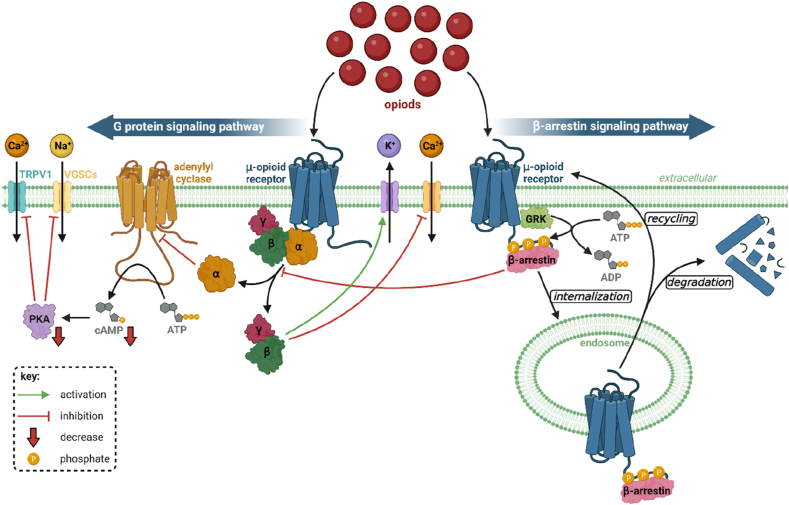
**Signaling pathways of the MOP receptors.** G protein signaling pathway (left side) is represented by fractionation of heterotrimer G_αβγ_ into G_α_ subunit and G_βγ_ heterodimer. The G_α_ subunit inhibits adenylyl cyclase which in turn inhibits production of cAMP and PKA. This results in inhibition of ion channels in the cellular membrane. Heterodimer G_βγ_ blocks calcium channels and opens potassium channels. These events result in reduction of excitability and nociception and result in analgesic effects. β-Arrestin signaling pathway (right side) is represented by GRK-mediated phosphorylation on C-terminus of the receptor, which results in recruitment of β-arrestin to the receptor. This results in desensitization and occasionally endocytosis of the receptor, commonly termed as receptor internalization. Both of these events decrease the responses to opioids, inducing tolerance and insufficient analgesia. MOP receptor on the endosome can be dephosphorylated and degraded by lysosomes or resensitized through trafficking back to the cellular membrane. Abbreviations: α, G_α_ subunit; β, G_β_ subunit; γ, G_γ_ subunit; ADP, adenosine diphosphate; ATP, adenosine triphosphate; cAMP, cyclic adenosine monophosphate; GRK, G protein-coupled receptor kinase; PKA, protein kinase A; TRPV1, transient receptor potential cation channel subfamily V member 1; VGSCs, voltage-gated sodium channels [[34], [35], [36]]. Created with BioRender.com.

Various ligands activating MOP receptors can provide different signaling pathways. Therefore, ligands can be classified according to biased agonism which refers to differences in downstream signaling pathways. Based on biased agonism, there are three major groups of MOP receptor ligands: non-biased ligands, G protein-biased ligands, and β-arrestin-biased lignads. Fentanyl is considered as β-arrestin-biased ligand [25]. The both, G protein and β-arrestin signaling pathways are shown in Fig. 2.

#### 2.3.3 Fentanyl acting on MOP receptors

**Fig. 3:**
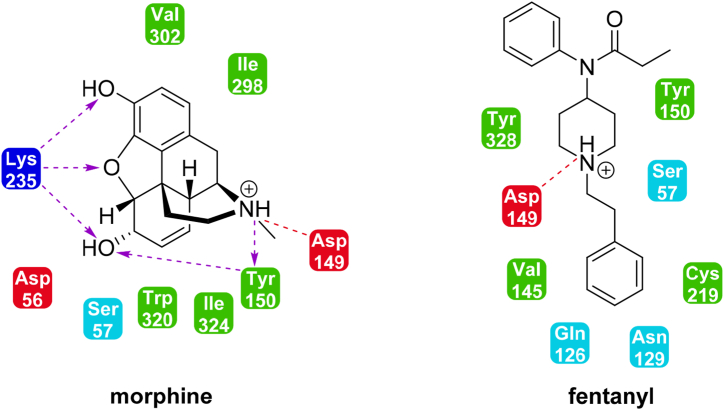
**Binding mode of morphine and fentanyl to MOP receptor.** Common and different notable interactions of the ligands with amino acid residues of the receptor [38].

Opioids acting on MOP receptors, such as morphine or fentanyl, are highly effective analgesics for treatment of severe pain. However, morphine exhibits different mode of action in comparison to fentanyl. In 2017, Schmid et al. reported that β-arrestin-biased ligands of MOP receptors, such as fentanyl, are more likely to develop respiratory depression at weak analgesic doses [23]. On the other hand, morphine primarily provides signaling via activation of G proteins, which is assumed to result in lowered respiratory depression [37]. Both, morphine and fentanyl, differ in their binding mode to the receptor and provide diverse intracellular conformational changes resulting in activation of diverse transmembrane (TM) helices. Ricarte et al. reported in 2021, that fentanyl provides a stronger effect on TM6 and TM7, while morphine acts predominantly on TM3 and TM5, on the basis of the molecular dynamics simulations of these ligands to human MOP receptor. Interestingly, a conformational change in TM6 is crucial for activation of MOP receptors as well as for G protein binding. These effects presumably contribute to enhance potency of fentanyl. The both ligands exhibit interactions with different amino acid residues of the MOP receptor. In particular, morphine interacts with Asp56, Lys235, Ile298, Val302, Trp320, and Ile324; while fentanyl interacts with Gln126, Asn129, Val145, Cys219, and Tyr328. However, interactions with Ser57, Tyr150, and Asp149 residues were found to be present for both morphine and fentanyl. Electrostatic interaction with Asp149 is essential for binding to MOP receptor. Interestingly, Lys235 exhibits interaction with any oxygen atom in the structure of morphine whereas interaction with oxygen in the structure of fentanyl is avoided. Notable interaction of morphine and fentanyl with MOP receptor are depicted in Fig. 3 [38]. One year earlier, in 2020, de Waal et al. evaluated mechanisms of fentanyl-mediated β-arrestin-biased signaling. The authors had found that aniline moiety of fentanyl pushes downward Met153 residue of MOP receptor. The study suggested that fentanyl-mediated displacement of Met153 is required for β-arrestin, but not for G protein coupling to the receptor. Aniline motif in fentanyl structure seems to play an important role in β-arrestin signaling [39].

#### 2.3.4 Pharmacokinetics of fentanyl

**Fig. 4:**
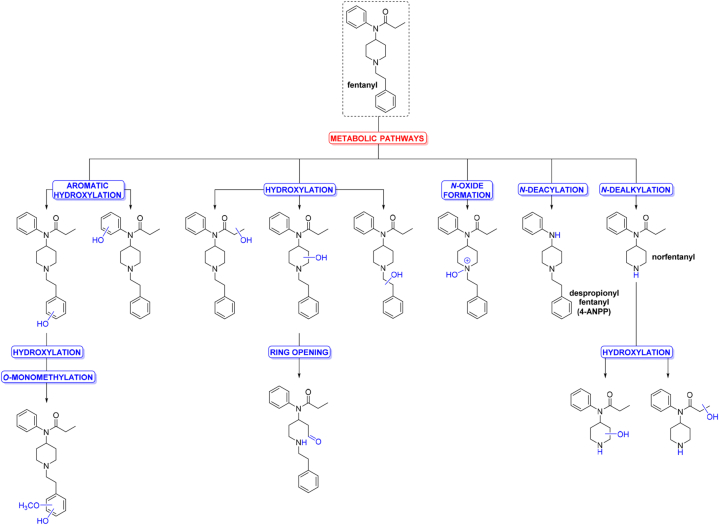
**Metabolic pathways of fentanyl.** Schematic view of metabolites detected in human. *N*-dealkylation represents the main metabolic pathway [44].

Fentanyl can be administered via different routes including intravenous, intramuscular, transdermal, intranasal, and intrathecal route, as skin patches or as a volatile nasal spray. Fentanyl is also available as a buccal soluble thin film, which can be dissolved in oral cavity [40]. Intravenously administered fentanyl is rapidly distributed from plasma into highly vascularized compartments. Then, after uptake in the systematic circulation, fentanyl is redistributed to muscle and fat tissue. After an initial equilibration of fentanyl follows its release from storage sites back to the plasma. Therefore, fentanyl has a long elimination half-life about 3–8 h. Transdermal route of fentanyl administration begins by its absorption through the skin which is followed by uptake into the cutaneous microcirculation and finally entering the systematic circulation. Fentanyl-containing products with rapid-onset are at first absorbed by highly vascularized oromucosal or nasal membranes, then entering the systematic circulation [41,42]. Fentanyl's volume of distribution is large (3.5–8 L/kg) and its clearance is relatively high (30–72 L/h) [13]. The main route of fentanyl metabolization occurs in the liver by cytochrome P450 isoenzymes 3A4 (CYP3A4) and 3A5 (CYP3A5). Fentanyl undergoes *N*-dealkylation to provide inactive norfentanyl as a main metabolite. Further metabolites of fentanyl are hydroxyfentanyl, norhydroxyfentanyl, and despropionyl fentanyl (4-ANPP), which are pharmacologically inactive and represent less than 1% of fentanyl metabolites in human liver microsomes (Fig. 4) [43]. The inactive metabolites and approximately 10% of non-metabolized fentanyl are excreted by the kidneys [41]. However, study reported by Ziesenitz et al., in 2015 suggested that other metabolic pathways can be involved in metabolism of fentanyl [43].

#### 2.3.5 Pharmacodynamics of fentanyl

Fentanyl can cross the blood-brain barrier (BBB) rapidly to produce analgesia within 1–2 min. Duration of analgesic action is 30–40 min; however, at high doses fentanyl can mediate a second peak of activity after its release from tissue stores [45]. One of the most serious adverse effects associated with fentanyl is respiratory depression, which can lead to the development of acute brain hypoxia, coma, and lethality [46]. However, most of the respiratory depression events associated with intravenously administered fentanyl in patients are not significant [13]. Common fentanyl side effects involve: headache, dizziness, drowsiness, nausea, vomiting, constipation, insomnia, swelling, sweating and other [47].

#### 2.3.6 Toxicity and abuse of fentanyl

Fentanyl is highly toxic compound with the median lethal dose (LD_50_) of 3.1 mg/kg in rats and 0.03 mg/kg in monkeys. Its LD_50_ in humans is unknown [48], but according to Drug Enforcement Administration (DEA), dose of 2 mg of fentanyl can be lethal depending on a person's body weight, tolerance and previous usage of the drug. Interestingly, drug trafficking organizations commonly distribute fentanyl by the kilogram scale, which has a potential to kill about 500 000 people [49].

Fentanyl was initially abused by medical staff, such as doctors (especially anesthesiologists), nurses, pharmacists, and support staff, who were easily exposed to controlled substances [50,51]. Fentanyl overdose may lead to death via serious anaphylactic response or cardiac issues. Overdose of fentanyl can progress to fatal respiratory depression within 2 min [52]. Blood concentration levels of around 7 ng/mL or higher are linked to deaths in which polydrug use was abused. The ingredients are mixed with other harmful street drugs such as cocaine or heroin and then sold to unaware users that are at a higher risk of overdose [53]. In comparison to heroin, fentanyl has a stronger opioid effect, the duration of action is shorter usually 1–2 h, and is more effective [54].

Since 2013, number of reported law enforcement encounters testing positive for fentanyl has increased dramatically in the United States (US). Early investigations provided strong evidence of an association between reported fentanyl encounters and fentanyl-involved overdose deaths. In addition, it has been found that most of fentanyl-involved overdose deaths were not associated with prescribed fentanyl but with illicitly-produced fentanyl. Moreover, fentanyl is often mixed with heroin or sold as heroin without providing information to the user. This fact significantly increases a potential treat of side effects or death. The current fentanyl crisis continues to expand across the US [55]. Indeed, the data regarding fentanyl fatal intoxications reported by the National Center for Health Statistics (NCHS) which is a part of the Centers for Disease Control and Prevention (CDC), the national public health agency of the US, manifest that deaths caused by illicitly-produced fentanyl sharply increased since 2013. In the US (28 states and District of Columbia), from July to December 2018, fentanyl was detected in 73.9% of overall 14 571 opioid-involved overdose deaths [56].

Treatment of fentanyl as well as other opioid overdose is usually mediated by naloxone, a synthetic derivative of oxymorphone [57]. Naloxone, also known as *N*-allyl noroxymorphone, acts as a non-selective and competitive opioid receptor antagonist. In addition to acute opioid overdose, naloxone further reverses respiratory and mental depression caused by opioids [58]. Naloxone onset relates to its rapid entry into the brain due to its high lipophilicity. The distribution half-life of drug is 4.7 min while the elimination half-life is about 65 min. Relative short duration time of naloxone can result in re-narconization effect of longer acting opioids causing reoccurrence of toxicity as well as respiratory depression [57]. Therefore, in long-term acting opioids repeated doses of naloxone are needed [59]. Compounds for treatment of fentanyl overdose seem to play a key role in upcoming years.

## 3 Analogs and derivatives of fentanyl

**Fig. 5:**
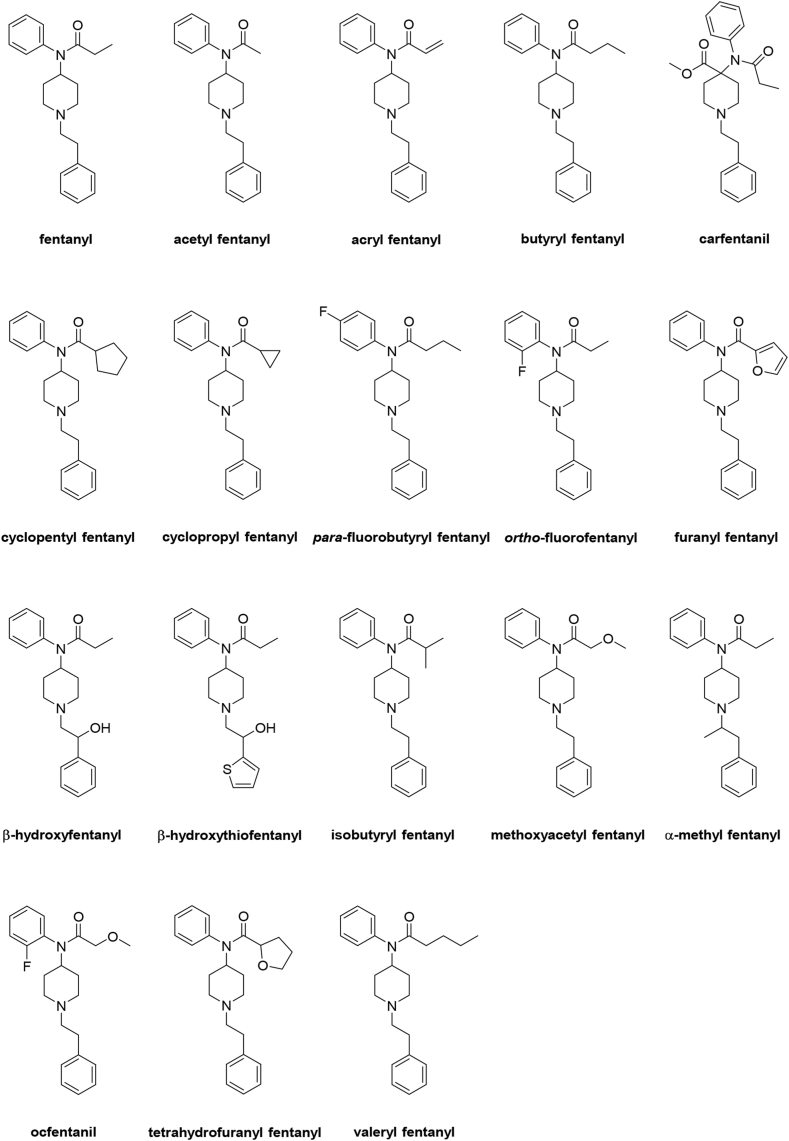
Chemical structures of fentanyl and selected fentanyl derivatives.

Based on above-mentioned synthesis of fentanyl [15], numerous fentanyl analogs and derivatives can be prepared in a similar manner by substituting some of the reagents used in the synthetic route. For example, upon substitution of aniline with *para*-fluoroaniline, *para*-fluorofentanyl can be prepared. Similarly, using 1-phenyl-2-bromopropane instead of initial 2-phenylethyl bromide in fentanyl synthesis provides α-methylfentanyl, a fentalog methylated on α-position of the phenethyl group. Variable fentanyl analogs, also called fentalogs, and derivatives, also called fentanyls, have been synthetized since 1964. These structural modifications involve replacement of *N*-benzene ring for other aromatic or non-aromatic group, substitution of amide group, replacement of propionyl group for other acyl substituents, modifications or substitution of piperidine ring, modifications or substitution of linker connecting piperidine ring with phenyl, replacement or modification of aromatic ring in phenethyl group and others. Structural modifications of fentanyl resulted in preparation of a large number of fentanyl derivatives, many of which are more effective than the parent compound and some have found an application in medicine [48]. Structures of notable fentalogs and fentanyl derivatives are shown in Fig. 5, summary of their selected activities is shown in Table 1, and their characterization is described below.

**Table 1:** Summary of recently reported binding Ki values to opioid receptors and antinociceptive activity of selected fentanyl analogs.

	Binding Ki values to opioid receptor [nM]			Binding ratio		Antinociceptive activity^a^	Potency ratio to	
	MOR	KOR	DOR	MOR/KOR	MOR/DOR	ED_50_ [mg/kg]	morphine	fentanyl
**morphine**	0.252	27.7	123	110	448	7.82	1.00	0.01
**fentanyl**	0.135	174	220	1290	1630	0.08	97.7	1.00
**acetyl fentanyl**	4.28	860	5700	531	1330	0.24	32.1	0.33
**acryl fentanyl**	0.133	110	102.23	827	769	–	–	–
**butyryl fentanyl**	0.405	215	317	531	783	0.11	57.7	0.59
**cyclopentyl fentanyl**	0.89	166	350	187	393	–	–	–
**cyclopropyl fentanyl**	0.088	36	59.4	409	1080	–	–	–
***para*-fluorobutyryl fentanyl**	–	–	–	–	–	0.91	8.61	0.09
***ortho*-fluorofentanyl**	–	–	–	–	–	0.03	224	2.30
**furanyl fentanyl**	0.0279	59.2	54	2120	1940	–	–	–
**isobutyryl fentanyl**	0.291	321	388	1100	1330	0.0768	102	1.04
**methoxyacetyl fentanyl**	0.560	907	1530	1620	2730	–	–	–
**tetrahydrofuranyl fentanyl**	0.95	741	1730	780	1820	–	–	–
**valeryl fentanyl**	2.16	467	1660	219	769	6.43	1.22	0.01
references	[21]					[60,61]		

a based on the warm-water tail-withdrawal test in mice.

### 3.1 Acetyl fentanyl

Acetyl fentanyl (CAS Registry Number: 3258-84-2), according to IUPAC nomenclature also termed as *N*-phenyl-*N*-[1-(2-phenylethyl)-4-piperidinyl]acetamide, is a derivative of fentanyl bearing *N*-acetyl group instead of *N*-propionyl motif. The first synthesis of acetyl fentanyl was reported by Janssen and Gardocki, in 1964 [62]. In comparison to fentanyl, acetyl fentanyl reveals reduced affinity to opioid receptors: Ki = 4.28 nM for MOP receptor, Ki = 860 nM for KOP receptor, Ki = 5700 nM for DOP receptor [21]. Analgesic potency ratio of acetyl fentanyl was evaluated in the study reported by Higashikawa and Suzuki, in 2008. The study revealed that peroral administration of acetyl fentanyl is 15.7, in comparison to analgesic potency of morphine; and 0.29, in comparison to potency of fentanyl [63]. More recently, in 2021, Walker et al. evaluated relative potency of subcutaneously administered acetyl fentanyl in comparison with two reference opioids, fentanyl and morphine, in male rats. In the antinociception assay, based on a measurement of tail withdrawal latencies, relative potency of acetyl fentanyl was approximately 4-fold higher in comparison with morphine, but approximately 31-fold lower in comparison with fentanyl. The authors further reported that acetyl fentanyl exhibits effects similar to fentanyl and morphine, moreover its effects were blocked by naltrexone, an opioid receptor antagonist which blocks opioid effects. These data suggest a similar abuse liability risk for acetyl fentanyl as for typical opioid agonists [64].

Symptoms of acetyl fentanyl intoxication or overdose are similar to other opioid analgesics [65]. Metabolism of acetyl fentanyl is expected to be similar to fentanyl metabolic pathway. Based on human hepatocyte and human urine metabolite studies, metabolism of acetyl fentanyl involves *N*-dealkylation providing acetyl norfentanyl, hydroxylation, aromatic dihydroxylation followed by *O*-monomethylation, deacetylation resulting in 4-ANPP as well as subsequent conjugations affording corresponding glucuronides or sulfates [66]. Acetyl fentanyl has become very popular among drug addicts and is commonly found as an admixture of street heroin [[67], [68], [69], [70]].

In the US (10 states), from July 2016 to December 2018, a total of 26 104 opioid-involved overdose deaths were reported. Within them acetyl fentanyl was the most commonly detected fentalog found in 8.3% (2178 cases) of deaths. In addition, deaths caused by acetyl fentanyl overdose significantly increased reaching the peak in December 2018, at the end of evaluated period, involving 179 cases. Later, from July to December 2018, a total of 14 571 opioid-involved overdose deaths in the US (28 states and Distinct of Columbia) were reported. Within this shorter period, acetyl fentanyl was again the most commonly detected fentalog associated with 16.2% (2363 cases) of deaths [56]. In Europe, between 2013 and September 2015, acetyl fentanyl was associated with 32 death cases in four Member States of the European Union (EU): Sweden (27 cases), Germany (2 cases), the United Kingdom (2 cases), and Poland (1 case). Interestingly, of the 32 deaths, one death occurred in 2013, two death cases in 2014, and 29 deaths occurred in 2015 (January–September), as reported by the European Monitoring Center for Drugs and Drug Addiction (EMCDDA) [71]. As reported later by Guerrieri et al., acetyl fentanyl fatal intoxications in Sweden, during 2015–2016, involved 34 cases [72]. In Russia, in 2012, acetyl fentanyl detected in post-mortem samples has been associated with 12 deaths [73]. The drug is often reaching heroin users, some of them administer acetyl fentanyl unknowingly [74].

### 3.2 Acryl fentanyl

Acryl fentanyl (CAS Registry Number: 82003-75-6), also known as acryloyl fentanyl or egyptenyl, according to IUPAC termed as *N*-phenyl-*N*-[1-(2-phenylethyl)piperidin-4-yl]prop-2-enamide, is an analog of fentanyl, in which *N*-propionyl motif is replaced by *N*-acryloyl group. Similar as fentanyl, acryl fentanyl is also highly lipophilic compound. The general synthetic pathway for illicitly-produced acryl fentanyl is most likely based on the synthetic method for preparation of fentanyl, in which 4-ANPP is *N*-acylated with acryloyl chloride [75]. Acryl fentanyl exhibits similar affinity to MOP receptor (Ki = 0.133 nM) in comparison to fentanyl. Moreover, its affinity to KOP receptor (Ki = 110 nM) and DOP receptor (Ki = 102 nM) are slightly higher than those of fentanyl [21]. Two earlier studies have investigated the analgesic effects of acryl fentanyl. In 1981, Zhu et al. reported that acryl fentanyl is about 170 more effective antinociceptive agent than morphine, upon intraperitoneal administration in rodent model [76]. Essawi et al. examined the antinociceptive activity of five fentanyl derivatives, including acryl fentanyl, using a mouse hot-plate analgesia assay. The analgesic effects of acryl fentanyl compared to fentanyl were shown to be more potent and longer acting at doses below 1 mg/kg upon intraperitoneal administration [77]. More recently, in 2022, Bilel et al. investigated *in vitro* and *in vivo* pharmaco-dynamic study of acryl fentanyl. *In vitro* studies revealed that acetyl fentanyl was able to activate MOP receptor with high potency, having the negative logarithm of the half maximal effective concentration (pEC_50_) of 8.13. However, the activity on the both KOP and DOP receptors was less potent, showing an incomplete concentration response curve. Acryl fentanyl promoted MOP receptor/G protein interaction with pEC_50_ of 7.87, and MOP receptor/β-arrestin 2 interaction with pEC_50_ of 6.76. *In vivo* experiments with intraperitoneal injected acetyl fentanyl performed in mice confirmed that the drug exhibits increased mechanical and thermal analgesia, and impairs motor and cardiorespiratory parameters [78].

*In vitro* and *in vivo* metabolic studies using human hepatocytes and urine specimens suggested a similar metabolic pathway of acryl fentanyl to those of fentanyl or acetyl fentanyl. The metabolic routes include *N*-dealkylation providing acryl norfentanyl, hydroxylation, aromatic dihydroxylation followed by *O*-monomethylation, deacetylation affording 4-ANPP and subsequent conjugations resulting in corresponding glucuronides [66]. While the parent drug would be most prevalent in the urine of overdose cases, metabolites of monohydroxylation and hydroxylation reactions were suggested as suitable analytical urine biomarkers in cases of non-overdose involving acryl fentanyl [79]. In the US (10 states), from July 2016 to December 2018, acryl fentanyl was associated with 1.4% (353 cases) of overdose deaths of a total 26 104 opioid-involved overdose deaths. During the period, deaths caused by acryl fentanyl reached maximum in February 2017 (122 cases). Interestingly, overdose deaths associated with acryl fentanyl significantly decreased from the middle of 2017 till the end of evaluated period (December 2018) [56]. In Europe, during April–September 2016, acryl fentanyl was associated with 42 death cases in four EU Member States: Sweden (39 cases), Denmark (1 case), Finland (1 case), and Latvia (1 case) [80].

### 3.3 Butyryl fentanyl

Butyryl fentanyl (CAS Registry Number: 1169-70-6), also known as butyrfentanyl, termed by IUPAC nomenclature as *N*-phenyl-*N*-[1-(2-phenylethyl)piperidin-4-yl]butanamide, is an analog of fentanyl, in which *N*-propionyl group is substituted by *N*-butyryl group [65]. Based on the synthesis of pervious fentalogs, synthetic pathway for preparation of butyryl fentanyl can follow the procedure for fentanyl preparation, except of the final step in which butyryl chloride or butyric anhydride is used for *N*-acylation of 4-ANPP. Butyryl fentanyl exhibits lowered affinity for MOP receptor (Ki = 0.405 nM) or only slightly reduced affinities for KOP (Ki = 215 nM) and DOP (Ki = 317 nM) receptors, in comparison to fentanyl [21]. Analgesic potency ratio of butyryl fentanyl to morphine is 7.0, while to fentanyl is 0.13, as based on study in mice after perorally administered drugs [63]. In 2018, Baumann et al. reported a study carried out in mice with subcutaneously administered butyryl fentanyl. The authors found that the drug was 31-fold more potent than morphine in the tail flick assay [81]. However, in the recent study, reported by Walker et al., in 2021, investigating effects of subcutaneously administered butyryl fentanyl in rats was found that its relative potency in the antinociception assay, based on the tail withdrawal latencies, was approximately 12-fold higher in comparison with morphine, 3-fold higher in comparison with acetyl fentanyl, and about 10-fold lower if compared with fentanyl. The effects of butyryl fentanyl were also blocked by naltrexone [64].

The metabolism of butyryl fentanyl was investigated by Kanamori et al. using fresh human hepatocytes isolated from a liver-humanized mouse model. As the main metabolite, butyryl norfentanyl formed by *N*-dealkylation from parent compound, which reached 37% of the initial amount of butyryl fentanyl after 48 h. Second and third most abundant metabolites were formed by hydroxylation of *N*-butyryl group are represented by ω-hydroxybutyryl fentanyl and (ω-1)-hydroxybutyryl fentanyl, respectively. Additional metabolites involve products of hydroxylation such as 4′-hydroxybutyryl fentanyl and β-hydroxybutyryl fentanyl, or aromatic dihydroxylation followed by *O*-monomethylation providing 4′-hydroxy-3′-methoxybutyryl fentanyl [82]. In the US (28 states and District of Columbia), during July–December 2018, butyryl fentanyl overdose deaths were associated with 86 cases, representing 0.6% of total 14 571 opioid-involved overdose deaths [56]. In Europe, butyryl fentanyl overdose deaths were also reported in Sweden, involving one death between 2015 and 2016 [72].

### 3.4 Carfentanil

Carfentanil (CAS Registry Number: 59708-52-0), named according to nomenclature of IUPAC as methyl 1-(2-phenylethyl)-4-(*N*-propanoylanilino)piperidine-4-carboxylate, is a derivative of fentanyl bearing methoxycarbonyl group at C4 position of piperidine ring. Several synthetic routes for carfentanil preparation using 4-piperidinone, as a starting material, are known [83]. Potency of carfentanil is about 10 000 higher than potency of morphine. In addition, carfentanil is about 30–100 more potent than fentanyl [84]. Carfenil represents the most potent approved opioid drug with application in veterinary medicine as an anesthetic, pain-killer, and as a drug immobilizing large animals [44,85]. In 2017, Wong et al. investigated *in vivo* effects of aerosolized carfentanil on physiological responses in mice. The authors evaluated clinical observations in animals exposed to carfentanil and control during exposure and at 24 h post-exposure period. Opioid-induced toxicity was observed in dose-dependent manner. Animals exposed to the drug exhibited significant decrease in minute volume. Moreover, observations during post-exposure period revealed decrease of mean arterial blood pressure, heart rate, and core body temperature. Post-exposure treatment with naloxone did not increase the minute volume, however, improved clinical signs of opioid-induced toxicity and duration of respiratory depression in animals exposed to carfentanil [86]. In 2019, Bergh et al. examined pharmacodynamic-pharmacokinetic relationships of subcutaneously administered carfentanil in rats. It has been found that carfentanil produces dose-dependent hypothermia and catalepsy, which is accompanied by nonlinear accumulation of the drug at high doses. Maximal concentration of the drug was observed after 15 min whereas its metabolite, norcarfentanil, reached the maximal concentration within 1–2 h. Plasma half-life for carfentanyl increased at higher doses. On the basis of these results the authors hypothesize that impaired clearance of the drug in human could by associated with life-threatening effects of this highly potent opioid agonist [87].

Biotransformation of carfentanil provides 12 different metabolites such as norcarfentanil, a metabolite provided by *N*-dealkylation. Norcarfentanil can be further hydroxylated or undergo ester hydrolysis. The parent drug can be hydroxylated at *N*-propionyl motif, phenethyl moiety or at the piperidine ring. Additional biotransformations involve *N*-oxidations or glucuronidation [44]. In the US (10 states), from July 2016 to December 2018, carfentanil was associated in 1724 cases of overdose deaths, representing 6.6% of overall opioid-involved overdose fatalities (26 104 cases). Deaths caused by cafentanil reached a peak twice within the observed period, first-time in September 2016 (87 deaths), second-time in April 2017 (235 cases). However, between April 2017 and December 2018, carfenil-caused deaths significantly decreased [56]. In Europe, from November 2016 to December 2017, an exposure to carfentanil was confirmed in 61 deaths. Fatalities were reported by the United Kingdom (31 cases), Lithuania (16 cases), Estonia (6 cases), Sweden (3 cases), Finland (2 cases). One case of death was reported by Belgium, Germany, and Norway. Moreover, the deaths reported by the United Kingdom occurred during February–June 2017 [88]. Interestingly, analysis of clothing and urine from Moscow theatre siege casualties revealed presence of carfentanil and remifentanil, an analgesic fentanyl derivative that is approved by FDA, used to incapacitate terrorists during the rescue operation [89].

### 3.5 Cyclopentyl fentanyl

Cyclopentyl fentanyl (CAS Registry Number: 2088918-01-9), according to IUPAC nomenclature known as *N*-phenyl-*N*-[1-(2-phenylethyl)piperidin-4-yl]cyclopentanecarboxamide, is an analog of fentanyl, in which *N*-propionyl group is replaced by *N*-cyclopentanecarbonyl group. Similar to above-mentioned fentalogs, synthesis of cyclopentyl fentanyl also can be accomplished upon reaction conditions for preparation of fentanyl; however, cyclopentanecarbonyl chloride have to be used for the final *N*-acylation step of 4-ANPP. Regarding binding affinity to opioid receptors, cyclopentyl fentanyl has subnanomolar affinity for MOP receptor (Ki = 0.89 nM), but the affinity is significantly lower in comparison to fentanyl. Its affinity for KOP receptor (Ki = 166 nM) is similar and for DOP receptor (Ki = 350) is reduced in comparison to fentanyl [21]. Metabolic pathway of cyclopentyl fentanyl evaluated by Astrand et al. revealed as the first and the third most abundant metabolites two monohydroxylated compounds, both hydroxylated on cyclopentyl ring, covering 31% and 11%, respectively, of the total metabolite area after 5 h. The second most abundant is the metabolite afforded by *N*-dealkylation, cyclopentyl norfentanyl, covering 29% of the total metabolite area. Additional metabolites are formed by hydroxylation, *N*-oxidation, *N*-deacetylation. Interestingly, no glucuronidated metabolites were observed for cyclopentyl fentanyl [90]. Cyclopentyl fentanyl intoxication has been confirmed in Sweden in 2016 [91]. However, data associated with cyclopentyl fentanyl overdose deaths are limited.

### 3.6 Cyclopropyl fentanyl

Cyclopropyl fentanyl (CAS Registry Number: 1169-68-2), according to IUPAC nomenclature termed as *N*-phenyl-*N*-[1-(2-phenylethyl)piperidin-4-yl]cyclopropanecarboxamide, is an analog of fentanyl, in which *N*-propionyl group is replaced by *N*-cyclopropanecarbonyl group. The first synthesis of cyclopropyl fentanyl was patented in 1965 by Paul Janssen [92]. Due to structural similarity with fentanyl differing only in *N*-acyl group, cyclopropyl fentanyl can by synthetized upon reaction conditions reported by Gupta et al., using cyclopropanecarbonyl chloride for the final 4-ANPP *N*-acylation reaction [15]. Cyclopropyl fentanyl exhibits increased binding affinities to opioid receptors than fentanyl. More specifically, Ki = 0.088 nM for MOP receptor, Ki = 36 nM for KOP receptor, and Ki = 59.4 nM for DOP receptor [21]. It is suggested, that cyclopropyl fentanyl is at least as potent as fentanyl [93]. In 2021, Bergh et al. evaluated pharmacokinetics and pharmacodynamics of subcutaneously administered cyclopropyl fentanyl in rats. In the study, cyclopropyl fentanyl exhibited dose-related increases of latency on hot plate with median effective dose (ED_50_) of 48 μg/kg and catalepsy with ED_50_ = 87 μg/kg. The highest dose of cyclopropyl fentanyl (300 μg/kg) resulted in long-lasting hypothermia. Cyclopropyl fentanyl reached maximal concentration in plasma within 15–28 min whereas maximal concentration of its metabolite, cyclopropyl norfentanyl, was observed after 45–90 min. Values of maximal concentration of the drug in plasma were similar with those measured in human non-fatal intoxication cases. The authors suggested an increased overdose risk of cyclopropyl fentanyl for unsuspecting users [94]. In 2023, Xie et al. investigated *in vitro* and *in vivo* effects of cyclopropyl fentanyl. *In vitro* assay examining MOP receptor coupling to G proteins revealed that cyclopropyl fentanyl acts as a full agonist with the half maximal effective concentration (EC_50_) = 8.6 nM, which was similar to fentanyl (EC_50_ = 10.3 nM). Effects of cyclopropyl fentanyl on KOP receptor (EC_50_ = 844 nM) and DOP receptor (EC_50_ = 1062 nM) were significantly lower. In the assay assessing MOP receptor-mediated recruitment of β-arrestin 2 recruitment cyclopropyl fentanyl (EC_50_ = 85.7 nM) showed higher potency than fentanyl (EC_50_ = 172.8 nM). *In vivo* studies, performed in mice, were conducted with drugs administered subcutaneously. Antinociceptive potency was evaluated in the tail flick assay. The potency of cyclopropyl fentanyl (ED_50_ = 0.04 mg/kg) was nearly equivalent to fentanyl (ED_50_ = 0.03 mg/kg) whereas potency of morphine (ED_50_ = 1.80 mg/kg) was 45-fold lower. Antinociceptive effects of cyclopropyl fentanyl were significantly reduced when animals were pretreatment with naloxone [95].

Metabolic profile of cyclopropyl fentanyl evaluated by Astrand et al., identified seven metabolites. As the major metabolite, afforded by *N*-dealkylation, cyclopropyl norfentanyl was determined, representing 79% of a total metabolite area after 5 h. The second most abundant metabolite was provided by hydroxylation of piperidine ring, covering 14% of a total metabolite area. Minor metabolites, covering 0.4–3.3% of total metabolite area, include metabolite afforded by aromatic monohydroxylation on phenethyl group, 4′-hydroxy-3′-methoxycyclopropyl fentanyl, one dihydrodiol or alternatively a ring-opened dehydroxylated metabolite, cyclopropyl fentanyl *N*-oxide, as well as one glucuronidated metabolite. Notably, metabolites oxidized on the cyclopropyl ring or metabolites formed by *N*-deacylation reaction were not observed [90]. In the US (10 states), from July 2016 to December 2018, cyclopropyl fentanyl was detected in 371 cases of drug overdose deaths, representing 1.4% of a total opioid-involved overdose deaths (26 104 cases). During the period, deaths caused by cyclopropyl fentanyl reached a peak in September 2017 (49 cases) [56]. In Europe, during June–October 2017, cyclopropyl fentanyl was associated with 60 deaths, as reported by the EMCDDA. The majority of these were reported by Sweden (59 cases), one death was reported by Norway [96]. In later period, during 2017–2018, two of the EU Member States (Sweden and the United Kingdom) reported 78 deaths caused by the drug. Overall, the drug has been detected in 6 EU Member States and Norway. Cyclopropyl fentanyl was detected in tablets among falsified benzodiazepines (e.g. counterfeit Xanax®) and opioid painkillers (e.g. counterfeit OxyContin®). This fact significantly increases the risk of poisoning in unsuspecting users [97].

### 3.7 *Para*-fluorobutyryl fentanyl

*Para*-fluorobutyryl fentanyl (CAS Registry Number: 244195-31-1), also commonly known as *para*-fluorobutyrfentanyl, according to IUPAC termed as *N*-(4-fluorophenyl)-*N*-[1-(2-phenylethyl)piperidin-4-yl]butanamide, is a fluorinated analog of butyryl fentanyl. Synthesis of *para*-fluorobutyryl fentanyl can be accomplished according to similar procedure for the preparation of butyryl fentanyl, in which aniline is substituted by *para*-fluoroaniline [98]. *In vivo* study of subcutaneously injected *para*-fluorobutyryl fentanyl based on the warm-water tail-withdrawal assay in mice was reported by Varshneya et al., in 2023. The drug exhibited a potent antinociceptive effect (ED_50_ = 0.91 mg/kg), having 0.09 potency ratio to fentanyl and 8.61 potency ratio to morphine. In addition to antinociception, *para*-fluorobutyryl fentanyl elicited significant dose-dependent hyperlocomotion and hypoventilation [60].

The data associated with *para*-fluorobutyryl fentanyl overdose deaths are limited, but several deaths related to the drug have been reported by the US and the EU. In the US, during 2016–2017, *para*-fluorobutyryl fentanyl was associated with 6 deaths [99,100]. In Europe, one death associated with the drug has been reported by Poland, in 2014 [101]; and also by Sweden, during 2015–2016 [72].

### 3.8 *Ortho*-fluorofentanyl

*Ortho*-fluorofentanyl (CAS Registry Number: 910616-29-4), according to IUPAC nomenclature named as *N*-(2-fluorophenyl)-*N*-[1-(2-phenylethyl)piperidin-4-yl]propenamide, is a fluorine analog of fentanyl bearing fluorine atom in *ortho*-position of *N*-phenyl ring. Synthesis of *ortho*-fluorofentanyl can be accomplished upon reaction conditions for the preparation of fentanyl as reported by Gupta et al. [15], in which aniline is replaced by *ortho*-fluoroaniline. In 2023, Varshneya et al. reported *in vivo* study performed in mice with subcutaneously injected *ortho*-fluorofentanyl. In the warm-water tail-withdrawal assay the drug showed a highly potent antinociceptive activity (ED_50_ = 0.03 mg/kg) with 2.30 potency ratio to fentanyl and 224 potency ratio to morphine. The study further confirmed eliciting effect of *ortho*-fluorofentanyl on hyperlocomotion and hypoventilation [60].

Metabolism of *ortho*-fluorofentanyl and two additional fluorofentanyl isomers has been investigated by Gundersen et al., using human hepatocyte study in comparison to authentic human urine samples, based on knowledge on the metabolism of similar fentalogs. Major metabolites were formed by *N*-dealkylation providing *ortho*-fluoronorfentanyl; hydroxylations at the alkyl chain, phenethyl ring, and/or piperidine ring, and methylation. The data also suggested a presence of *ortho*-fluorofentanyl *N*-oxide [102]. Data associated with *ortho*-fluorofentanyl overdose deaths are limited. Moreover, some intoxications caused by fluorinated fentanyls using general name suc as fluorofentanyl without specification of the isomerism. However, fatal exposure to *ortho*-fluorofentanyl has also been reported [103].

### 3.9 Furanyl fentanyl

Furanyl fentanyl (CAS Registry Number: 101345-66-8), according to IUPAC nomenclature termed as *N*-phenyl-*N*-[1-(2-phenylethyl)piperidin-4-yl]furan-2-carboxamide, is an analog of fentanyl, in which *N*-propionyl group is substituted by *N*-(2-furoyl) group. The compound first appeared in a patent published in 1986 [104]. According to synthesis of previous fentalogs, preparation of furanyl fentanyl can be accomplished upon reaction conditions for synthesis of fentanyl [15], using 2-furoyl chloride for *N*-acylation of 4-ANPP. Affinities of furanyl fentanyl to opioid receptors are higher than those of fentanyl. The drug also exhibits higher affinity to MOP receptor (Ki = 0.0279 nM), than to KOP receptor (Ki = 59.2 nM) or DOP receptor (Ki = 54 nM) [21]. In 2022, a study evaluating *in vitro* and *in vivo* effects of furanyl fentanyl was reported by Bilel et al. *In vitro* evaluation of the drug revealed its activity on MOP receptor (pEC_50_ = 7.93), however agonistic effects on KOP and DOP receptors were not observed. Furanyl fentanyl promoted interaction of MOP receptor/G protein (pEC_50_ = 8.66), whereas MOP receptor/β-arrestin 2 interaction was not mediated with the drug. *In vivo* evaluation in mice with intraperitoneal injected furanyl fentanyl revealed its increased mechanical and thermal analgesic effects. However, in comparison with fentanyl, acryl fentanyl, and ocfentanyl; effects of furanyl fentanyl on cardiorespiratory and motor functions were lowered [78].

Metabolic pathway of furanyl fentanyl combining human hepatocyte and human urine metabolite evaluations has been reported by Watanabe et al., in 2017. As the most abundant metabolite, 4-ANPP, a product of *N*-deacylation has been detected, which can be further hydroxylated on *N*-phenyl ring, piperidine ring or on the aliphatic linker connecting piperidine core and phenyl. The second most dominant biotransformation involved epoxidation of furan followed by hydration providing dihydrodiol metabolite. The dihydrodiol can be further hydroxylated or *N*-dealkylated. *N*-dealkylation of furanyl fentanyl provided furanyl norfentanyl. Further biotransformations involve furanyl ring opening and subsequent carboxylation or formation of glucuronide and sulfate conjugates [66]. In the US, between 2015 and July 2016, 128 deaths associated with furanyl fentanyl have been reported [105]. Later, from July 2016 to December 2018, in 10 states of the US furanyl fentanyl was associated with 497 deaths, representing 1.9% of a total 26 104 opioid-involved overdose deaths. Within this period, a peak of furanyl fentanyl overdose deaths (100 cases) was detected in January 2017. However, from the middle of 2017 to the end of evaluated period, overdose deaths caused by the drug significantly decline [56]. In Europe, from November 2015 to February 2017, furanyl fentanyl has been associated with 23 deaths in 6 countries involving Sweden (12 cases), Estonia (4 cases), Germany (4 cases), Finland (1 case), Norway (1 case), and the United Kingdom (1 case). In all of the cases, furanyl fentanyl was analytically confirmed in post-mortem samples [105].

### 3.10 β-Hydroxyfentanyl

β-Hydroxyfentanyl (CAS Registry Number: 78995-10-5), also known as fentanol, according to IUPAC termed as *N*-[1-(2-hydroxy-2-phenylethyl)piperidin-4-yl]-*N*-phenylpropanamide, is an analog of fentanyl hydroxylated in β-position of phenethyl group. The metabolism of β-hydroxyfentanyl has been *in vitro* evaluated by Li et al. in human and rat liver microsomes. Norfentanyl, a product of *N*-dealkylation, has been determined as the major metabolite. The authors further established a method to determine four fentanyl-based analytes including β-hydorxyfentanyl in rat urine [106]. β-Hydroxyfentanyl was sold as an illicit drug in 1980s. Data regarding β-hydorxyfentanyl overdose deaths are unknown. However, recently Hendrickson et al. reported a case of intoxication by the drug in 22-year-old woman, which ingested a small quantity of a white powder containing β-hydroxyfentanyl (120 mg/g). The patient became unresponsive and apneic within minutes. The patient awoke after intramuscularly administered naloxone and was transported to an emergency and discharged to home after few hours. β-Hydroxyfentanyl was detected in patient's serum at the concentration of 6.5 ng/mL [107].

### 3.11 β-Hydroxythiofentayl

β-Hydroxythiofentanyl (CAS Registry Number: 1474-34-6), termed as *N*-[1-(2-hydroxy-2-thiophen-2-ylethyl)piperidin-4-yl]-*N*-phenylpropanamide according to IUPAC nomenclature, is a derivative of β-hydorxyfentanyl, in which benzene core of β-hydroxyphenethyl motif is substituted by heterocyclic thiophene core. The metabolism of β-hydroxythiofentanyl involves *N*-dealkylation providing norfentanyl as a detectable metabolite. Fatalities associated with β-hydroxythiofentanyl were also reported in 2015 in the US (Florida), in 7 cases [108]. Similar to β-hydroxyfentanyl, data regarding overdoses associated specific with β-hydroxythiofentanyl are also limited.

### 3.12 Isobutyryl fentanyl

Isobutyryl fentanyl (CAS Registry Number: 119618-70-1), also known as isobutyrfentanyl, termed according to IUPAC as 2-methyl-*N*-phenyl-*N*-[1-(2-phenylethyl)piperidin-4-yl]propenamide, is an analog of fentanyl, in which *N*-propionyl group is replaced by *N*-isobutyryl group. Due to modification in *N*-acyl motif, synthesis of the fentalog can be based on modified synthesis of fentanyl reported by Gupta et al. [15] using isobutyryl chloride for the final *N*-acylation. Isobutyryl fentanyl exhibits lowered affinities to opioid receptors in comparison to fentanyl: Ki = 0.291 nM for MOP receptor, Ki = 321 nM for KOP receptor, and Ki = 388 nM for DOP receptor [21]. Analgesic potency ratio of isobutyryl fentanyl to morphine is 6.9, while to fentanyl is 0.13 as based on study in mice after perorally administered drugs. These results are almost identical to analgesic potency of butyryl fentanyl [63]. Data regarding isobutyryl fentanyl overdose deaths are limited. Due to numerous structural isomers, similar mass spectral fragmentation patterns, and chromatographic resolution, the identification and quantification of fentanyl analogs such as isobutyryl and butyryl fentanyl can prove to be difficult. This fact increases a demand for a development of novel analytical methods in human samples. In 2018, Kahl et al. reported a method with ability to distinguish the isomers of fentalogs such as isobutyryl and butyryl fentanyl [109]. Similar methods can expand our knowledge about deaths associated with fentanyl analogs.

### 3.13 Methoxyacetyl fentanyl

Methoxyacetyl fentanyl (CAS Registry Number: 101345-67-9), termed according to IUPAC nomenclature as 2-methoxy-*N*-phenyl-*N*-[1-(2-phenylethyl)piperidin-4-yl]acetamide, is a derivative of fentanyl bearing *N*-methoxyacetyl motif instead of *N*-propionyl group. Its synthesis can be accomplished via preparation of 4-ANPP upon reaction conditions reported by Gupta et al. [15] using methoxyacetyl chloride for the final *N*-acylation. The affinities of methoxyacetyl fentanyl to opioid receptors are lowered than those of fentanyl. The highest affinity was reported for MOP receptor (Ki = 0.560 nM), while affinities for KOP (Ki = 907 nM) and DOP (Ki = 1530 nM) receptors were significantly lowered [21]. Based on the ED_50_ values, potency ratio of methoxyacetyl fentanyl to fentanyl is 0.3 [110]. Recent study from 2020, reported by Vasudevan et al., assessed MOP receptor activation with methoxyacetyl fentanyl via recruitment of mini-G_i_ protein (GTPase domain of G_αi_ subunit) and β-arrestin 2 to evaluate its analgesic effects. Agonistic effect of the drug resulted in recruitment of both, mini-G_i_ protein (pEC_50_ = 6.612) and β-arrestin 2 (pEC_50_ = 6.791) [111].

The metabolic pathway of methoxyacetyl fentanyl, based on the *in vitro* and *in vivo* metabolic profiles, has been investigated by Mardal et al., in 2018. The authors identified 10 metabolites involving *N*-dealkylation, *O*-demethylation, amide hydrolysis as well as combinations thereof. The main observed metabolites are *O*-demethylated metabolite and 4-ANPP, the product of amide hydrolysis. Further metabolic pathways involve monohydoxylations and dihydroxylations at the phenethyl motif or *N*-phenyl ring. In addition, *O*-glucuronidation of *O*-demethylated metabolite was also observed [44,112]. In the US (28 states and District of Columbia), during July–December 2018, deaths associated with methoxyacetyl fentanyl were reported in 85 cases, representing 0.6% of total opioid-involved overdose deaths (14 571 cases) [56]. In Europe, from December 2016 to February 2018, methoxyacetyl fentanyl has been associated with 13 deaths in four EU Member States involving Sweden (6 cases), the United Kingdom (5 cases), Belgium (1 case), and Czech Republic (1 case) [113].

### 3.14 α-Methyl fentanyl

α-Methyl fentanyl (CAS Registry Number: 79704-88-4), according to IUPAC nomenclature termed as *N*-phenyl-*N*-[1-(1-phenylpropan-2-yl)piperidin-4-yl]propenamide, is an analog of fentanyl bearing a methyl group attached to α-position of phenethyl motif. Synthesis of α-metyl fentanyl can be achieved upon reaction conditions as reported in 1974 by Van Bever et al. for a preparation of α-substituted 3-methyl fentanyl derivatives [84]. The analgesic potency of α-methyl fentanyl is similar to the potency of fentanyl. The ratio of analgesic potency of the drug is 1.1 to fentanyl and 56.9 to morphine [63]. Metabolic pathways of α-methyl fentanyl were investigated in biological samples of fatal intoxication cases [114], as well as in rat urine [115,116]. The biotransformation of α-methyl fentanyl provides norfentanyl, as a product of *N*-dealkylation that was observed as a major product in study on rats; ω-hydroxy and (ω-1)-hydroxy norfentanyls, as products of norfentanyl hydroxylation on the propionyl amide moiety; ω-hydroxy and (ω-1)-hydroxy α-methyl fentanyls, as products of α-methyl fentanyl hydroxylation on the propionyl amide moiety; despropionyl α-methyl fentanyl, as a product of amide hydrolysis; 4′-hydroxy-α-methyl fentanyl, as a product of aromatic hydroxylation; 4′-hydroxy-ω-hydroxy α-methyl fentanyl, as a product of aromatic hydroxylation followed by hydroxylation on the propionyl amide motif [44]. Fatal intoxications caused by α-methyl fentanyl overdose were reported in early 1980s [114]. The drug was associated with 15 deaths [117].

### 3.15 Ocfentanil

Ocfentanil (CAS Registry Number: 101343-69-5), also known as *ortho*-fluoromethoxyacetyl fentanyl, termed according to IUPAC nomenclature as *N*-(2-fluorophenyl)-2-methoxy-*N*-[1-(2-phenylethyl)piperidin-4-yl]acetamide, is an analog of methoxyacetyl fentanyl which is fluorinated at *ortho*-position of *N*-phenyl group. Its synthesis can be accomplished upon reaction conditions for synthesis of methoxyacetyl fentanyl, in which *ortho*-fluoroaniline is used instead of aniline [118]. The drug was patented by Huang et al., in 1986 [104]. The analgesic potency ratio of ocfentanil to fentanyl is 2.3 [110]. *In vitro* study reported in 2022 by Bilel et al. confirmed that ocfentanil acts on MOP receptors (pEC_50_ = 7.78) whereas evaluation of its activity on both, KOP and DOP receptors, showed an incomplete concentration response curve. Agonostic effect of ocfentanyl on MOP receptor resulted in recruitment of G protein (pEC_50_ = 8.09) as well as β-arrestin 2 (pEC_50_ = 7.28). *In vivo* analysis of interperitoneally administered ocfentanyl in mice revealed increased mechanical and thermal analgesic effects of the drug. Impairment of motor and cardiorespiratory parameters was also associated with administration of ocfentanyl [78].

Metabolic pathaways of ocfentanil have been investigated by Allibe et al., using human liver microsomes, as well as from post-mortem samples of a fatal intoxication case. The main metabolite was formed upon *O*-demethylation to provide *O*-demethyl ocfentanil. Additional metabolites observed in the study are represented by mono-hydroxylated ocfentanil, hydroxylated *O*-demethyl ocfentanil, and a glucuronide of the *O*-demethyl ocfentanil. Except the glucuronide, all metabolites were observed in post-mortem samples of blood and bile [119]. Interestingly, commonly observed metabolites of fentanyls such as products of *N*-dealkylation or amide hydrolysis have not been detected. This suggests that the pathways providing these metabolites play only a minor role [44]. In Europe, fatalities associated with ocfentanil were reported in two countries: Belgium (1 case) and Switzerland (1 case) [120,121]. Ocfentanil belongs to non-controlled fentalogs [119].

### 3.16 Tetrahydrofuranyl fentanyl

Tetrahydrofuranyl fentanyl (CAS Registry Number: 2142571-01-3), named according to IUPAC nomenclature as *N*-phenyl-*N*-[1-(2-phenylethyl)piperidin-4-yl]oxolane-2-carboxamide, is a saturated analog of furanyl fentanyl bearing *N*-(2-tetrahydrofuroyl) moiety. Synthesis of tetrahydrofuranyl fentanyl can be achieved under conditions for a synthesis of fentanyl reported by Gupta et al., using tetrahydro-2-furancarbonyl chloride for the final *N*-acylation of 4-ANPP [15]. Tetrahydrofuranyl fentanyl exhibits lowered affinities to opiate receptors than fentanyl or furanyl fentanyl, with highest affinity to MOP receptor (Ki = 0.95 nM), followed by KOP (Ki = 741 nM) and DOP (Ki = 1730 nM) receptors [21]. Evaluation of effectivity on the MOP receptor performed by Hassanien et al. showed that tetrahydrofuranyl fentanyl (EC_50_ = 390 nM) is about 12-fold less potent than fentanyl (EC_50_ = 32 nM) [122].

Investigation of tetrahydrofuranyl fentanyl metabolic pathway has been investigated by Krotulski et al. in human liver microsomes and confirmed by the metabolic profile of post-mortem blood and urine specimens. The authors identified 7 metabolites, most notably tetrahydrofuran norfentanyl, as the product of *N*-dealkylation and two metabolites, as the products of monohydroxylation. Further biotransformation involves amide hydrolysis resulting in 4-ANPP, which can be further monohydroxylated. One proposed metabolite is represented by the product of internal hydrolysis [123]. In the US, during 2016–2017, two deaths were reported in association with tetrahydrofentanyl (New Jersey and Wisconsin). In Europe, from September 2016 to March 2017, the drug caused 14 fatal intoxications in Sweden [124].

### 3.17 Valeryl fentanyl

Valeryl fentanyl (CAS Registry Number: 122882-90-0), also known as pentanoyl fentanyl, according to IUPAC nomenclature termed as *N*-phenyl-*N*-[1-(2-phenylethyl)piperidin-4-yl]pentanamide, is an analog of fentanyl in which *N*-propionyl group is replaced by *N*-pentanoyl motif. The synthesis of valeryl fentanyl can be accomplished similarly to above-mentioned *N*-acyl substituted fentalogs, using valeryl chloride for *N*-acylation of 4-ANPP [15]. In comparison to fentanyl, the drug expresses lowered affinities to opioid receptors: Ki = 2.16 nM for MOP receptor, Ki = 467 nM for KOP receptor, and Ki = 1660 nM for DOP receptor [21]. In 2019, Varshneya et al. reported *in vivo* study evaluating effects of valeryl fentanyl on locomotion and antinociception in mice. It has been found, that valeryl fentanyl elicits antinociceptive activity (ED_50_ = 6.43 mg/kg) in the warm-water tail-withdrawal assay, having 1.22 potency ratio to morphine and 0.0125 potency ratio to fentanyl. Effect of naltrexone 11.9-fold decreased antinociceptive effect of the drug. Interestingly, valeryl fentanyl did not exhibit effects on locomotion up to 100 mg/kg [61]. *In vitro* and *in vivo* effects of valeryl fentanyl were investigated by Xie et al., in 2023. *In vitro* study based on the coupling of MOP receptor with G protein showed that the drug acts as a partial agonist of the receptor (EC_50_ = 179.8 nM). However, valeryl fentanyl is more effective in recruitment of β-arrestin 2 to MOP receptor (EC_50_ = 77.6 nM). *In vivo* study performed in mice, based on the tail flick assay, showed that subcutaneously administered valeryl fentanyl (ED_50_ = 4.0 mg/kg) is 133-fold less potent than fentanyl and about 2-fold less potent than morphine. In addition, the authors carried out molecular dynamics simulations of the drug in active site of MOP receptors and concluded that the alkyl chain of valeryl fentanyl cannot be well accommodated at this position. This finding is in agreement with the data obtained from *in vitro* and *in vivo* studies [95].

Cooman et al. investigated metabolic pathway of valeryl fentanyl by comparison of *in vitro* human liver microsome model with an *in vivo* zebrafish model. The authors discovered 19 metabolites involving valeryl norfentanyl, as a product of *N*-dealkylation, and hydroxylation as the major metabolic pathway. The product of amide hydrolysis, 4-ANPP, was also detected. The study demonstrated similar metabolic profile of valeryl fentanyl in human liver microsome and zebrafish model [125]. In the US (Michigan), intoxications by valeryl fentanyl were associated with 13 deaths. The drug has been found in all of the 13 cases in post-mortem samples [126].

## 4 Abuses and accidents associated with fentanyl and its derivatives

### 4.1 Fentanyl derivatives as designer drug

As described above, synthesis of fentanyl is easily achievable in laboratory conditions [15]. Application of derivatized structural components, upon similar or modified reaction conditions, can provide a plenty of structurally altered compounds. Indeed, more than 1400 fentanyl derivatives have been described in the scientific and patent literature [127,128]. Fentanyls and other opioids, as opioid receptor agonists, provide numerous therapeutic and adverse effects. Opioids are powerful analgesics and can also produce euphoria. Prolonged and repeated usage of opioids may result to drug tolerance, dependence, and drug addiction [129]. Due to synthetic accessibility and pharmacological profile of fentanyl derivatives, these compounds represent an excellent example of designer drugs. A designer drug can be defined as a structural analog or derivative designed to mimic pharmacological effects of original controlled substance, with aim to avoid its classification as an illicit drug. Fentanyl derivatives, as designer drugs, are commonly also termed as illicitly manufactured fentanyls (IMFs). In recent years, many of IMFs found their use as street illicit drugs. Due to an extreme potency, IMFs are often mixed with other known illicit drugs, such as heroin, and the counterfeit mixture is sold to unsuspecting users under original name. Thus, the counterfeit drug is usually more potent, cheaper, more addictive, and more dangerous in comparison to original drug. Moreover, IMFs increasingly passed into counterfeit pills resembling alprazolam, oxycodone, or other prescription drugs and are expanding into new markets in the US [130].

### 4.2 Fentanyl derivatives and opioid epidemic

**Fig. 6:**
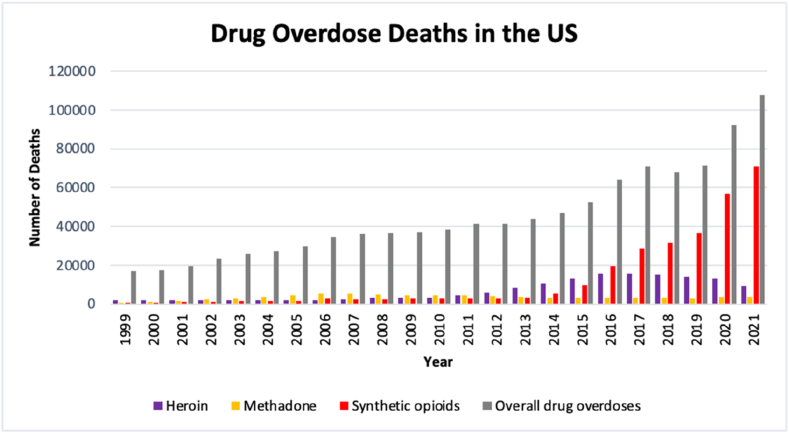
**Drug overdose deaths in the US between 1999 and 2021.** Overall drug overdose deaths (grey) are shown along with selected drugs such as heroin (purple), methadone (yellow), and synthetic opioids involving fentanyl and excluding methadone (red). Source: CDC. Statistics during 1999–2014 are based on the NCHS report (December 2020) [133]. Statistics during 2015–2021 are based on the NCHS updated report (September 2, 2022) [3].

The opioid epidemic, sometimes also referred to as the opioid crisis, is characterized by the growing number of misuses, overuses, and deaths associated with opioid drugs, in the US. Background of the opioid can be dated back to the 1980s, when pain increasingly became recognized as a problem that required adequate treatment. Although opioids were prescribed earlier, mainly to patients after surgery or people suffering from advanced cancer, an idea was to provide safer and less addictive opioids as painkillers. During 1980s to early 1990s, opioid prescriptions increased gradually however, in the middle of 1990s, when new opioid drugs were introduced by pharmaceutical companies, prescriptions of opioids increased and the drugs to treat chronic pain become widespread [131]. According to the CDC, the opioid epidemic involves three significant increasements in the number of cases, commonly termed as “waves”. The first wave started in the late 1990s, as the result of increased overdose deaths caused by prescriptions of opioid to treat pain. The second wave started in 2010, as the result of increased overdose deaths caused by heroin. The third wave started in 2013, as the result of increased overdose deaths caused by synthetic opioids [132]. Of the three waves, the third wave is the deadliest with increasing number of overdose deaths, since 2012 [133]. In Fig. 6, the numbers of drug overdose death cases in the US, during 1999–2021, are shown.

In 1999, synthetic opioids represented 4.3% of total drug overdose deaths. Over the next 10 years, the percentage of synthetic opioids in total drug-caused fatalities almost doubled. During the second wave of the opioid epidemic deaths associated with synthetic opioids slightly decreased from 3007 cases in 2010–2628 cases in 2012. However, since 2013 to the present, the number of overdose deaths caused by synthetic opioids is considerably increasing. For instance, in 2017, synthetic opioid-involved overdose deaths represented 40.5% of the 70 699 overall drug overdose deaths reported in the US. Four years later, in 2021, synthetic opioids were associated with 71 074 deaths, representing 66.1% of overall drug overdose deaths in the US. More than 107 000 people died of a drug overdose in 2021, in the US. Of the synthetic opioids, fentanyl manufactured illicitly is primarily responsible for this rapid increase. However, overdose deaths caused by IMFs also play a significant role [3,56,133]. Reported drug overdose deaths in the US, between 1999 and 2021, are summarized in Table 2. In addition to the US, synthetic opioids contribute to overdose deaths in other countries. In Europe, usage of opioid prescriptions for pain management has also increased in the last two decades. In spite of the fact that several cases have been reported in different European countries, investigation of four EU Member States (France, Germany, Netherlands, and the United Kingdom) by van Amsterdam et al. demonstrated that there is no evidence of a current or emerging opioid crisis in these EU Member States. However, authorities should remain vigilant [134].

**Table 2:** **Summarized drug overdose deaths in the US between 1999 and 2021.** Source: CDC. Statistics during 1999–2014 are based on the NCHS report (December 2020) [133]. Statistics during 2015–2021 are based on the NCHS updated report (September 2, 2022) [3].

Year	Heroin		Methadone		Synthetic opioids		Overall drug overdose (death cases)
	death cases	percentage of overall deaths^a^	death cases	percentage of overall deaths^a^	death cases	percentage of overall deaths^a^	
**1999**	1960	11.6%	784	4.7%	730	4.3%	16 849
**2000**	1842	10.6%	986	5.7%	782	4.5%	17 415
**2001**	1779	9.2%	1456	7.5%	957	4.9%	19 394
**2002**	2089	8.9%	2358	10.0%	1295	5.5%	23 518
**2003**	2080	8.1%	2972	11.5%	1400	5.4%	25 785
**2004**	1878	6.8%	3845	14.0%	1664	6.1%	27 424
**2005**	2009	6.7%	4460	15.0%	1742	5.8%	29 813
**2006**	2088	6.1%	5406	15.7%	2707	7.9%	34 425
**2007**	2399	6.7%	5518	15.3%	2213	6.1%	36 010
**2008**	3041	8.3%	4924	13.5%	2306	6.3%	36 450
**2009**	3278	8.9%	4696	12.7%	2946	8.0%	37 004
**2010**	3036	7.9%	4577	11.9%	3007	7.8%	38 329
**2011**	4397	10.6%	4418	10.7%	2666	6.4%	41 340
**2012**	5925	14.3%	3932	9.5%	2628	6.3%	41 502
**2013**	8257	18.8%	3591	8.2%	3105	7.1%	43 982
**2014**	10 574	22.5%	3400	7.2%	5544	11.8%	47 055
**2015**	13 051	24.8%	3309	6.3%	9610	18.3%	52 623
**2016**	15 551	24.3%	3390	5.3%	19 500	30.5%	63 938
**2017**	15 593	22.1%	3210	4.5%	28 659	40.5%	70 699
**2018**	15 102	22.3%	3047	4.5%	31 525	46.5%	67 850
**2019**	14 116	19.8%	2756	3.9%	36 603	51.5%	71 130
**2020**	13 253	14.3%	3561	3.9%	56 894	61.5%	92 478
**2021**	9259	8.6%	3695	3.4%	71 074	66.1%	107 521
**Σ**_**(1999-2021)**_	152 557	14.6%	80 291	7.7%	289 557	27.8%	1 042 534

a of overall drug overdose deaths.

### 4.3 Moscow theater hostage crisis

On October 23, 2002 at 21:45, a group of 53-armed terrorist seized 979 hostages in Dubrovka Theater in Moscow [135]. The crisis was lasting for several days and an effort to find a peaceful resolution failed [136]. Early in the morning, at around 5:00 on October 26, 2002, Russian authorities decided to use an aerosol of potent incapacitating agents against the terrorist, with aim to narcotize them and save hostages. The aerosol was pumped into the building and drew the attention of both, the rebels and the hostages. Some of the rebels succumbed to the effect of the aerosol. However, some rebels were situated in areas where the concentration of aerosol was lower, others donned gas masks that had been brought along for the attack. Rebels protected from the aerosol effects responded by firing at the Russian forces, which prolonged the rescue operation. At around 7:00, only 3 terrorists reminded alive in the building. As the result of the hostage crisis, 130 hostages died, of them 125 died due to exposure to the aerosol [89,135]. Further investigation revealed several misconducts during the rescue operation that contribute to a high number of victims. First, until the beginning of rescue operation it was not revealed that aerosol would be used, therefore the medical care was inadequately prepared. Second, even when information that aerosol had been used was provided to medical staff, it was not specified type of the aerosol, which resulted in delayed, inappropriate or often even not provided treatment. Third, in spite that the information about naloxone as an antidote was provided to emergency responders, delayed informing resulted in vastly limited supply of the antidote. Fourth, empty syringes found in the theater indicate that the members of Russian forces had been armed with the antidote, however it seems likely that this supply was rapidly depleted. Moreover, due to the overwhelming number of hostages affected by the aerosol, utility of the antidote supply at the scene was limited [135]. At first it was not clear what specific substance was contained in the aerosol that Russian security forces had released into the theatre. On October 30, 2002, Russian Health Minister, Yuri Shevchenko, identified the aerosol as a fentanyl derivative that fell into the category of non-lethal medical substances not prohibited by the Chemical Weapons Convention (CWC) [137]. In 2012, based on the analysis of clothing and urine from casualties, British scientists found that the aerosol contained carfentanil, one of the most dangerous derivatives of fentanyl, used as an anesthetic for large animals. In addition, presence of rimefentanil, an analgesic fentanyl derivative that is approved for treatment in human, was also reported [89].

### 4.4 Fentanyl derivatives as a potential chemical weapon

Incapacitating agents are compounds acting on the CNS and produce temporary physiological and/or mental effects in affected individuals. Effects of the agents may persist for hours or day, but victim usually do not require medicinal treatment. Although, the treatment may accelerate recovery [138]. These compounds may be administered through contaminated food and drinks or applied as aerosols. The compounds were originally designed to reduce effectiveness of soldiers [139]. An ideal incapacitating agent with rapid onset represents a valuable counter-terrorist weapon [140]. In 1990s, fentanyl and its derivatives were investigated in the US by the Department of Defense as possible incapacitating agents. However, complications associated with the margin of safety, an optimal dosage to incapacitate but not to kill, were never resolved and the project was abandoned [141]. An effort to develop incapacitating agent from fentanyl derivatives has also been undertaken by Soviet/Russian militaries [142]. On the other side, fentanyl derivatives in hands of terrorists represent a significant threat. Considering potency and accessibility of fentanyl derivatives poses a potential risk of their misuse as chemical warfare agents. Especially, the accessibility of fentanyl derivatives can play an important role in their misuse as a potential chemical weapon, because development of traditional chemical weapons, such as sarin, requires significant chemical engineering capabilities, specialized equipment and resources [141]. Furthermore, as seen in Moscow theater hostage crisis, inappropriate application of compounds such toxic as fentanyl derivatives may cause a tragedy, even with the compounds in good hands [89]. This fact exposed a grey area between lethal and non-lethal weapons and triggered a discussion on limits of the CWC regarding the potential use of fentanyl as a weapon of mass destruction (WMD) [142]. Very recently, in September 2022, the 18 state attorneys general demand the president of the US to take decisive action in classification of fentanyl as a WMD in response to the record increase in overdose deaths related to the lethal substance nationwide and the potential for deliberate misuse. Fentanyl is now (September 2022) the number one case of death for adults aged 18–45, in the US [143].

## 5 Conclusion

Fentanyl and its derivatives are anesthetics used for the treatment of severe to moderate pain [48]. In addition to fentanyl, only three fentanyl derivatives have been approved for medicinal use in human, represented by alfentanil, remifentanil, and sufentanil [11]. Fentanyl and its derivatives are highly addictive synthetic opioids. The drugs are applied in the form of injection, transdermal patch, or lozenges. The availability of synthetic opioids increased strongly when transdermal fentanyl patches were introduced into clinical practice [144]. Fentanyl patches are now produced in huge quantities and their abuse pose a serious health risk [145]. The application of transdermal patches needs adequate knowledge and expertise in terms of the patient to avoid undesired side effects of the drug [146]. Inappropriate or illegal use of fentanyl patches may cause fatal poisoning [147]. Cases of death following intravenous administration of fentanyl extracted from fentanyl patches are documented [14,[145], [146], [147], [148], [149], [150]]. In addition, the accessibility of fentanyl and its derivatives play a significant role in the increasing number of deaths associated with the drugs. Fentanyl manufactured illicitly, as well as other IMFs represent a serious global problem of nowadays. These highly potent opioids are often mixed with other narcotics available on the illegal market with aim to improve potency, reduce price and increase addiction of the user. Fentanyl or IMFs thus may be sold on the illegal market without users' knowledge [55]. Moreover, IMFs increasingly passed into counterfeit pills in the US, representing a significant treat due to easier availability [130]. In 2021, more than 107 000 drug overdose death cases have been reported in the US, 66.1% of them were associated with synthetic opioids, mainly represented by fentanyl or its derivatives. These drugs now represent leading cause of death for adults aged 18–45, in the US [3,143]. This phenomenon related to synthetic opioid abuse has been referred to as the “opioid epidemic” [151]. In Europe, fentanyl and its derivatives are also common on the illegal market but the number of deaths is not as high as in the US. It seems likely that the risk of opioid epidemic in Europe is not anticipated yet, however, the authorities should remain vigilant [134].

Nowadays, illicit fentanyl and its derivatives represent a highly dangerous threat to public health. These compounds are presented as a different variety of products often disguised as heroin, other drugs of abuse, or counterfeit pharmaceuticals. New analogs are constantly being produced in order to bypass controls performed by authorities. This represents a significant treat to unaware consumers. Due to insufficient research on fentanyl, it is difficult to obtain a comprehensive and up-to-date list of all fentanyl analogs, their properties and drug metabolites. Several analytical techniques can be used for detection of fentanyl and a plenty of its derivatives [152]. However, due to a simple method of preparation novel derivatives of fentanyl are still emerging. Therefore, detection of a wide range of chemical scaffolds represents a constant challenge. Despite the fact that opioid epidemic is a well-known phenomenon, further education and information of the public can improve understanding of the danger associated with misuse of fentanyl and its derivatives.

## Ethics statement

Review and approval by an ethics committee was not needed for this study because this was a literature review and no new data were collected and analysed.

## Additional information

No additional information is available for this paper.

## Data availability statement

Data will be made available on request.

## CRediT authorship contribution statement

**Jiri Patocka:** Writing – original draft, Methodology, Conceptualization. **Wenda Wu:** Writing – original draft, Data curation. **Patrik Oleksak:** Writing – original draft, Methodology, Data curation. **Romana Jelinkova:** Writing – original draft, Investigation. **Eugenie Nepovimova:** Writing – review & editing, Funding acquisition, Formal analysis. **Lenka Spicanova:** Writing – original draft, Methodology. **Pavlina Springerova:** Writing – original draft, Supervision. **Suliman Alomar:** Writing – review & editing, Funding acquisition, Formal analysis. **Miao Long:** Writing – review & editing, Supervision, Formal analysis. **Kamil Kuca:** Writing – review & editing, Supervision, Conceptualization.

## Declaration of competing interest

The authors declare that they have no known competing financial interests or personal relationships that could have appeared to influence the work reported in this paper.
